# The Relation Between Passively Collected GPS Mobility Metrics and Depressive Symptoms: Systematic Review and Meta-Analysis

**DOI:** 10.2196/51875

**Published:** 2024-11-01

**Authors:** Yannik Terhorst, Johannes Knauer, Paula Philippi, Harald Baumeister

**Affiliations:** 1 Department of Clinical Psychology and Psychotherapy Institute of Psychology and Education University Ulm Ulm Germany; 2 Department of Psychology Ludwig Maximilian University of Munich Munich Germany; 3 German Center for Mental Health (DZPG), Partner Site Munich-Augsburg Munich Germany; 4 Department of Clinical Child and Adolescent Psychology and Psychotherapy Institute of Psychology University of Wuppertal Wuppertal Germany

**Keywords:** smart sensing, digital phenotyping, depression, GPS, global positioning system, meta-analysis, mobile phone, depressive symptoms, smartphone, systematic review, depressive disorders, treatment, mental disorder, mental health, wearable

## Abstract

**Background:**

The objective, unobtrusively collected GPS features (eg, homestay and distance) from everyday devices like smartphones may offer a promising augmentation to current assessment tools for depression. However, to date, there is no systematic and meta-analytical evidence on the associations between GPS features and depression.

**Objective:**

This study aimed to investigate the between-person and within-person correlations between GPS mobility and activity features and depressive symptoms, and to critically review the quality and potential publication bias in the field.

**Methods:**

We searched MEDLINE, PsycINFO, Embase, CENTRAL, ACM, IEEE Xplore, PubMed, and Web of Science to identify eligible articles focusing on the correlations between GPS features and depression from December 6, 2022, to March 24, 2023. Inclusion and exclusion criteria were applied in a 2-stage inclusion process conducted by 2 independent reviewers (YT and JK). To be eligible, studies needed to report correlations between wearable-based GPS variables (eg, total distance) and depression symptoms measured with a validated questionnaire. Studies with underage persons and other mental health disorders were excluded. Between- and within-person correlations were analyzed using random effects models. Study quality was determined by comparing studies against the STROBE (Strengthening the Reporting of Observational studies in Epidemiology) guidelines. Publication bias was investigated using Egger test and funnel plots.

**Results:**

A total of k=19 studies involving N=2930 participants were included in the analysis. The mean age was 38.42 (SD 18.96) years with 59.64% (SD 22.99%) of participants being female. Significant between-person correlations between GPS features and depression were identified: distance (*r*=–0.25, 95% CI –0.29 to –0.21), normalized entropy (*r*–0.17, 95% CI –0.29 to –0.04), location variance (*r*–0.17, 95% CI –0.26 to –0.04), entropy (*r*=–0.13, 95% CI –0.23 to –0.04), number of clusters (*r*=–0.11, 95% CI –0.18 to –0.03), and homestay (*r*=0.10, 95% CI 0.00 to 0.19). Studies reporting within-correlations (k=3) were too heterogeneous to conduct meta-analysis. A deficiency in study quality and research standards was identified: all studies followed exploratory observational designs, but no study referenced or fully adhered to the international guidelines for reporting observational studies (STROBE). A total of 79% (k=15) of the studies were underpowered to detect a small correlation (*r*=.20). Results showed evidence for potential publication bias.

**Conclusions:**

Our results provide meta-analytical evidence for between-person correlations of GPS mobility and activity features and depression. Hence, depression diagnostics may benefit from adding GPS mobility and activity features as an integral part of future assessment and expert tools. However, confirmatory studies for between-person correlations and further research on within-person correlations are needed. In addition, the methodological quality of the evidence needs to improve.

**Trial Registration:**

OSF Registeries cwder; https://osf.io/cwder

## Introduction

Depressive disorders are one of the most prevalent mental disorders worldwide. The global prevalence rate is estimated to be 4.4% [1,2]. Associated consequences of depression are severe not only for affected individuals (eg, globally, 43 million years lived with disability in 2017) but also for the economy and society [3-5]. Costs are even higher and long-term health consequences are more severe if the disorder is not properly diagnosed or treated [6,7]. To target the global burden caused by depression and to initiate effective treatment, timely diagnosis is a key bottleneck in health care [8-10].

Various diagnostic approaches like structured clinical interviews [11], clinician-rated screening scales [12,13], and self-report screening assessments [14] are available. However, their reliability and accuracy are often hindered by social desirability, recall, or confirmation biases [15-17]. Therefore, augmentations and extensions of existing approaches by objective data sources could potentially make an important contribution to the improvement in the diagnostic process of depression and other mental disorders.

Facing an ever-growing digitalization in daily living, the availability of various sensor data in smartphones, wearable devices, cars, and smart home devices may provide a way toward objective diagnosis in a timely manner and sensor-informed diagnostic support tools for clinical personnel [18-21]. In the context of medical apps, the approach of using sensor data to infer mental health is referred to as smart sensing or digital phenotyping [18,19]. In short, raw data from software (eg, screen status of the smartphone [on or off]) and hardware-based (eg, GPS coordinates) sensors are processed to derive higher-level features (eg, total smartphone usage time, total distance, and circadian rhythm), which are then linked to clinical symptoms or diagnoses [18,19].

Earlier studies highlight the potential of smart sensing [22]: For instance, Opoku Asare et al [23] found an area under the curve (AUC) of the receiver operating curve (ROC) of 94.69% to 99.06% in the prediction of depression status (depressed or not depressed) using a supervised machine learning model on behavioral markers from the smartphone (ie, app usage, screen usage, and network usage features). Furthermore, studies have shown that smart sensing data can improve prediction and increase explained variance over self-report ratings such as ecological momentary assessments [24,25]. In line with these findings, various descriptive reviews underline the potential shown in the present literature [26-33].

While there is a broad array of sensors in the context of depression, in particular, GPS-based mobility and activity features (eg, total distance, places visited, and time spent at home) could be promising candidates for objective and unobtrusive measurement of core symptoms of depression such as reduced activity, fatigue, loss of functioning, and diminished interest [24,27,34,35]. Individual studies find medium to high correlations between depressive symptoms and GPS mobility and activity features (eg, number of location clusters: *r*=–0.38, circadian movement: *r*=–0.34, home stay: *r*=0.22 [34]; eg, [24,35-37]). However, the heterogeneity in the findings and in the methodology between studies is substantial [27,31]. In addition, a mean sample size of 23.1 (SD 27.9) was reported in the systematic review of Cornet and Holden [26], clearly highlighting the issue of underpowered trials in the field. Meta-analytical evidence is highly needed to provide an estimate of the relationship between GPS mobility and activity features and depressive symptoms to determine their applicability for clinical settings and to transfer research from exploratory investigations to confirmatory studies. However, to date, no quantitative meta-analysis has been conducted on the associations between GPS mobility and activity features and depression.

Therefore, this study systematically reviewed and meta-analyzed the current evidence on the associations between GPS mobility and activity features and depression. Besides, core aspects of study quality (eg, adherence to international reporting guidelines, preregistrations, presence of a-priori and post hoc power analysis [31,38,39]) and the potential of publication bias in the field were investigated. Research questions are (1) What is the pooled between-person correlation of GPS mobility and activity features and depression severity? (2) What is the pooled within-person correlation of GPS mobility and activity features and depression severity? (3) To what extent are international reporting guidelines (ie, STROBE [Strengthening the Reporting of Observational studies in Epidemiology] [38]) reflected in the studies? and (4) Are small study effects as an indicator of potential publication bias observable in the literature?

## Methods

### Study Design

This study is a systematic review and meta-analysis. All procedures have been preregistered in the open science framework under trial registration link. Reporting is conducted in accordance with the updated PRISMA (Preferred Reporting Items for Systematic Reviews and Meta-Analyses) guidelines for the reporting of systematic reviews [40] (Multimedia Appendix 1).

### Search and Eligibility Criteria

Given the interdisciplinary nature of the field, we searched MEDLINE, PsycINFO, Embase, CENTRAL, ACM, IEEE Xplore, PubMed, and Web of Science. The database-specific search strings can be found in Multimedia Appendix 2. After the removal of duplicates by automatic tools of the databases, all identified articles were screened in a two-stage process: (1) title and abstract screening and (2) full-text screening, both performed by 2 independent reviewers (YT and JK). If duplicates were encountered in this screening process, they were removed by hand. In addition to the database search, we searched all reference lists of the included studies for further eligible studies. This search and inclusion process was started on December 6, 2022 (database searches from December 06 until June 13), and completed by March 24, 2023 (last reference search). Any disagreements between the reviewers were resolved in discussions.

To be eligible, the following inclusion criteria had to be met: (1) any kind of GPS sensor data collection, (2) the data were collected by a wearable device such as smartphone or smartwatch, (3) an assessment of depressive symptoms was conducted either by self-report or by clinical diagnostic scales, and (4) reported outcomes included correlations between the collected GPS sensor data and depressive symptoms. Studies that (1) comprised participants younger than 18 years of age or (2) included participants with disorders other than depression (eg, bipolar disorder) were excluded.

### Measured Variables and Coding

All data were extracted by 2 independent reviewers (JK and YT). The following study characteristics and empirical data points were obtained.

#### Study Characteristics

To describe the included studies, we extracted the authors’ names, publication year, measures of depression (eg, Patient Health Questionnaire-9 [PHQ-9]), GPS-sensor features (ie, mobility and activity features such as total distance), study design, study setting, sensing framework (app name) and sample characteristics (age, gender, population, and country).

#### Quantitative and Empirical Data

For the statistical analysis, correlation coefficients (both between-person and within-person) and sample size were extracted (see further analysis details below). Between-person correlations were defined as the interindividual associations between 2 variables (eg, do individuals with higher time spent at home tend to show higher depression?). Within-person correlations were defined as intraindividual associations of 2 variables across time (eg, does an individual tend to show higher depression in weeks with more time spent at home?). Corresponding authors of eligible studies with insufficiently reported information for meta-analysis (eg, reporting of *P* values without correlation coefficients) were contacted for the missing information (ie, repeated email containing a study description and extraction template for the needed information). Furthermore, all included studies were compared against international guidelines for reporting observational studies (STROBE [38]). In addition, the presence of a preregistration and a priori or post hoc power analysis were rated as additional criteria (assessment of research standards and small study effects are provided below). The rating was performed independently by 2 researchers (JK and YT). Disagreements were resolved in discussion. Study designs other than exploratory, observational, intensive, longitudinal designs were planned to be compared against corresponding guidelines of the EQUATOR Network but were not present in the included studies.

### Statistical Analysis

#### Meta-Analysis of Correlation

We conducted a random effects meta-analysis of correlations. Pooling was based on inverse variance weighting [41]. Maximum likelihood was used as the estimator. Following the Cochrane handbook for meta-analysis, the minimum required number of studies reporting on the correlations between a feature (eg, home stay) and depression to run meta-analysis was 2 [42]. For all significance tests and range of CI, was set to 5%. Heterogeneity of effect sizes was evaluated by *I*^2^ [43]. For heterogeneity, we defined an *I*^2^ of 25% as the threshold for low, 50% for moderate, and 75% for high heterogeneity in the present meta-analysis. 95% CI and prediction intervals were calculated along the pooled correlation estimates.

#### Assessment of Research Standards and Small Study Effects

As a general criterion for the adherence to international reporting guidelines, we assessed the reference and adherence to the STROBE guidelines [38] of the included observational studies. The STROBE checklist was rated for each study by 2 independent researchers (YT and JK). Disagreements were resolved in discussion.

Facing the replication crisis in research [39,44], we additionally investigated the percentage of preregistrations and the presence of a priori and post hoc power calculation. Potential publication bias was investigated using (1) funnel plots and (2) Egger test [45,46]. Egger test was only conducted in analyses with at least 10 studies [47]. Funnel plots display the effect sizes reported in studies (x-axis) in respect to the SE (y-axis). The core assumption is that in case of no publication bias, all points in the funnel plot should be distributed equally around an average effect with the high precision studies (low SE) at the top of the figure and closely around the average, and studies with low precision (high SE) should be broadly distributed around the average effect. In contrast to this, a scenario with publication bias (eg, small studies are only published if they show high effects, while large studies with lower effects are published either way due to the number of participants) would lead to an asymmetric funnel plot, where an association between SE and reported effect is visible. Capitalizing on this idea, Eggert test is a regression model investigating whether the SE is significantly influencing the average reported effect. Both asymmetries in funnel plots and significant Eggert tests indicate potential publication bias, and hence, potentially biased meta-analytically results (eg, systematic overestimation of the true effect due to unpublished high SE studies with no effects). For a more in-depth introduction, refer previously published studies (eg, [41]).

#### Software

All analyses were conducted in R (R Foundation for Statistical Computing). *Meta* was used as the core package in the analysis [48]. For an overview of all loaded packages and versions, see Multimedia Appendix 3. Both the analysis code and the dataset containing the correlations are available under CC-BY 4.0 license at the Open Science Framework (Multimedia Appendix 4).

## Results

### Study Selection

**Figure 1:**
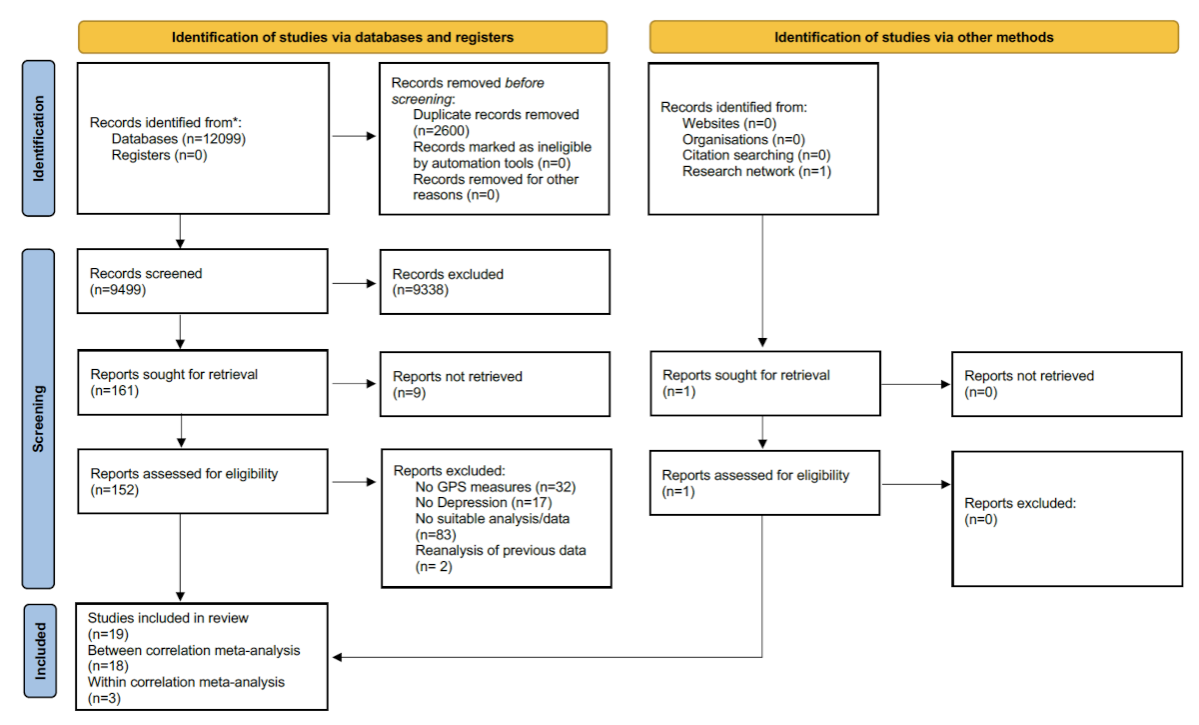
PRISMA (Preferred Reporting Items for Systematic Reviews and Meta-Analyses) flowchart. Records identified from (*) MEDLINE, PsycINFO, Embase, CENTRAL, ACM, IEEE Xplore, PubMed, and Web of Science.

We identified a total of k=9499 unique records in the systematic literature search. One additional study was screened and included after being forwarded by researchers contacted due to insufficient data reported in an identified study. In the following screening and inclusion process conducted by 2 independent reviewers (YT and JK), k=19 studies were finally included [24,34-37,49-62]. In total, 2 of the studies were eligible for between- and within-person correlation meta-analysis [34,53]. For further details, please refer to the PRIMSA flowchart in Figure 1.

### Study Characteristics

Combined, the included studies comprised a total of N=2930 participants. The sample sizes ranged from a minimum of n=18 to a maximum of n=1046 (mean 154.21, SD 235.54; median 72, IQR 83). The mean age across all included studies was 38.42 (SD 18.96) years. The average percentage of female participants was mean 59.64% (SD 22.99%). Of the k=19 included studies, k=8 (42%) studies explicitly targeted students and young adults (eg, recruitment at universities), k=3 (16%) studies recruited from the general public, k=5 (26%) studies used nontailored online and social media recruitment, k=2 (11%) studies targeted older adults, and k=1 (5%) study provided no further information on the recruitment strategies and targeted population. Please refer to Table 1 for further details on the descriptive characteristics of the studies.

A total of k=13 unique sensing frameworks were applied among the included studies. PurpleRobot was the most frequently used framework (k=3/19, 16%). Based on the sensed GPS data, the studies reported n=12 distinct activity and mobility features. The number of unique studies per activity and mobility feature ranged from k=1 to k=14 and a combined sample size of n=69 to n=2287 per feature. A summary of the features can be found in Table 2 for between-person features and Table 3 for within-person features.

**Table 1:** Main characteristics of included studies.

Reference	Country	N	Depression scale	Age (y), mean (SD)	Software	Period	Target population	Severity
Boukhechba et al [56]	United States	72	DASS-21^a^	19.8 (2.4)	Sensus	2 weeks	Students and young adults	3.5
Canzian and Musolesi [57]	United Kingdom	28	PHQ-8^b^	31 (—^c^)	Mood- Traces	Average 71 days	General public	—
Currey and Torous [37]	United States	147	PHQ-9^d^	—	mind-LAMP	4 weeks	Students and young adults	—
DeMasi and Recht [58]	United states	33	BDI^e^	—	—	8 weeks	Students and young adults	12.7
Di Matteo [59]	Canada	71	PHQ-8	30.6 (9.4)	Logger	2 weeks	Nontailored online and social media recruitment	9.1
Farhan et al [62]	United States	79	PHQ-9	—	Life-Rhythm	—	Students and young adults	—
Giannouli et al [60]	Germany	69	GDS^f^	69.5 (4.9)	Ufall & custom app	1 week	Older adults	1.39
Lu et al [50]	United States	103	QIDS^g^	—	Life-Rhythm & Fitbit	—	Students and young adults	—
MacLeod et al [49]	Canada	121	CES-DC^h^	18 (2.76)	Custom app	2 weeks	Students and young adults	32.6
Stamatis et al^i^ [61]	United States	1046	PHQ-8	40.9 (—)	LifeSense	16 weeks	Nontailored online and social media recruitment	9.14
Moshe et al [24]	Various European	55	DASS-21	42.8 (11.6)	AWARE	30 days	Nontailored online and social media recruitment	3.8
Nickels et al [51]	United States	379	PHQ-9	—	—	12 weeks	People who are or are not depressed	—
Saeb et al (2015a) [35]	United States	28	PHQ-9	28.9 (10.1)	Purple-Robot	2 weeks	General public	5.6
Saeb et al (2015b) [54]	—	18	PHQ-9	—	Purple-Robot	2 weeks	Nontailored online and social media recruitment	5.8
Saeb et al (2016) [34]	United States	48	PHQ-9	—	Student-Life	10 weeks	Students and young adults	—
Saeb et al (2017) [55]	United States	206	PHQ-9	39.3 (10.3)	Purple-Robot	6 weeks	General public	9.7
Tung et al [52]	Canada	54	GDS	72.6	VALMA	3 days	Older adults	—
Wang et al [36]	United States	83	PHQ-8 and PHQ-4^j^	20.1 (—)	Student-Life	9 weeks	Students and young adults	6.1
Zhang et al [53]	Netherlands, Spain, United Kingdom	290	PHQ-8	—	—	2 weeks	—	—

^a^DASS-21: Depression Anxiety and Stress Scale.

^b^PHQ-8: Patient Health Questionnaire-8.

^c^Not applicable.

^d^PHQ-9: Patient Health Questionnaire-9.

^e^BDI: Beck’s Depression Inventory.

^f^GDS: Geriatric Depression Scale.

^g^QIDS: Quick Inventory of Depression Symptomatology.

^h^CES-DC: Center for Epidemiological Studies–Depression.

^i^Stamatis et al [61] provided additional data on depression and GPS mobility metrics obtained from the LifeSense project for this meta-analysis. For further details on the LifeSense project, please refer to [61,63,64].

^j^PHQ-4: Patient Health Questionnaire-4.

**Table 2:** Between-person features.

GPS feature	Definition	Studies	Frequency	Total N	Value, mean (SD)
Homestay	Percentage of time spent home	Boukhechba et al [56], Currey and Torous [37], Di Matteo [59], Farhan et al (Android) [62], Farhan et al (iOS) [62], Lu et al (Android) [50], Lu et al (iOS) [50], Stamatis et al^a^ [61], Moshe et al [24], Nickels et al [51], Saeb et al (2015a) [35], Saeb et al (2015b) [54], Saeb et al 2016 [34], Saeb et al (2017) [55]	14	2216	158.29 (266.4)
Location variance	Variance of latitude and longitude values	Currey and Torous [37], DeMasi and Recht [58], Di Matteo [59], Farhan et al (Android) [62], Farhan et al (iOS) [62], Lu et al (Android) [50], Lu et al (iOS) [50], Stamatis et al [61], Moshe et al [24], Nickels et al [51], Saeb et al (2015a) [35], Saeb et al (2015b) [54], Saeb et al (2016) [34], Zhang et al [53]	14	2287	163.36 (276.1)
Entropy	Distribution of time spent at different location clusters	Currey and Torous [37], Di Matteo [59], Farhan et al (Android) [62], Farhan et al (iOS) [62], Lu et al (Android) [50], Lu et al (iOS) [50], Stamatis et al [61], Moshe et al [24], Nickels et al [51], Saeb et al (2015a) [35], Saeb et al (2015b) [54], Saeb et al (2016) [34], Zhang et al [53]	13	2254	173.38 (284.71)
N clusters	Number of unique location clusters	Currey and Torous [37], Di Matteo [59], Farhan et al (Android) [62], Farhan et al (iOS) [62], Lu et al (Android) [50], Lu et al (iOS) [50], Stamatis et al [61], Nickels et al [51], Saeb et al (2015a) [35], Saeb et al (2015b) [54], Saeb et al (2016) [34], Wang et al [36], Zhang et al [53]	13	2282	175.54 (283.85)
Norm entropy	Entropy normalized by the number of location clusters	Di Matteo [59], Farhan et al (Android) [62], Farhan et al (iOS) [62], Lu et al (Android) [50], Lu et al (iOS) [50], MacLeod et al [49], Stamatis et al [61], Moshe et al [24], Saeb et al (2015a) [35], Saeb et al (2015b) [54], Saeb et al (2016) [34], Zhang et al [53]	12	1849	154.08 (290.46)
Distance	Total distance between coordinates	Di Matteo [59], Lu et al (Android) [50], Lu et al (iOS) [50], MacLeod et al [49], Stamatis et al [61], Moshe et al [24], Saeb et al (2015a) [35], Saeb et al (2015b) [54], Saeb et al (2016) [34], Tung et al [52], Zhang et al [53]	11	1824	165.82 (301.64)
Speed moving	Speed at GPS data point collection	Farhan et al (Android) [62], Farhan et al (iOS) [62], Lu et al (Android) [50], Lu et al (iOS) [50], Stamatis et al [61], Saeb et al (2016) [34]	6	1266	211 (409.36)
Time moving	Time spent in moving states in percentage	Farhan et al (Android) [62], Farhan et al (iOS) [62], Lu et al (Android) [50], Lu et al (iOS) [50], MacLeod et al [49], Zhang et al [53], Di Matteo [59], Saeb et al (2015a) [35], Saeb et al (2015b) [54], Saeb et al (2016) [34]	10	748	74.8 (81.54)
Circadian movement	Amount of energy in frequency periods (eg, 30 min) based on least-squares spectral analysis	Di Matteo [59], Stamatis et al [61], Saeb et al (2015a) [35], Saeb et al (2015b) [54], Saeb et al (2016) [34]	5	1201	240.2 (450.9)
Life space area	Convex hull of GPS coordinates	Giannouli et al [60]	1	69	69 (—^b^)
Maximum action range	Longest (straight-line) distance away from home	Giannouli et al [60]	1	69	69 (—)

^a^Stamatis et al [61] provided additional data on depression and GPS mobility metrics obtained from the LifeSense project for this meta-analysis. For further details on the LifeSense project, please refer to [61,63,64] ^b^Not applicable.

^b^Not applicable.

**Table 3:** Within-person features.

GPS feature	Definition	Studies	Frequency	Total N	Value, mean (SD)
Distance	Total distance between coordinates	Canzian and Musolesi [57], Saeb et al (2016) [34], Zhang et al [53]	3	356	118.67 (148.46)
Entropy	Distribution of time spent at different location clusters	Saeb et al (2016) [34], Zhang et al [53]	2	328	164 (178.19)
Location variance	Variance of latitude and longitude values	Saeb et al (2016) [34], Zhang et al [53]	2	328	164 (178.19)
N clusters	Number of unique location clusters	Saeb et al (2016) [34], Zhang et al [53]	2	328	164 (178.19)
Norm entropy	Entropy normalized by the number of location clusters	Saeb et al (2016) [34], Zhang et al [53]	2	328	164 (178.19)
Circadian movement	Amount of energy in frequency periods (eg, 30 min) based on least-squares spectral analysis.	Saeb et al (2016) [34]	1	38	38 (—^a^)
Homestay	Percentage of time spent home	Saeb et al (2016) [34]	1	38	38 (—)
Speed moving	Speed at GPS data point collection	Saeb et al (2016) [34]	1	38	38 (—)
Time moving	Time spent in moving states in percentage	Saeb et al (2016) [34], Zhang et al [53]	2	328	164 (178.19)

^a^Not applicable.

### Meta-Analysis: Between Person Correlations

Distance was the mobility and activity feature most strongly associated with depressive symptoms, with a meta-analytically pooled between-person correlation of *r*=–0.25 (95% CI –0.29 to –0.21), followed by normalized entropy (*r*=–0.17, 95% CI –0.29 to –0.04), location variance (*r*=–0.17, 95% CI –0.26 to –0.06), entropy (*r*=–0.13, 95% CI –0.23 to –0.04), number of clusters (*r*=–0.11, 95% CI –0.18 to 0.03), and home stay (*r*=0.10, 95% CI 0.00 to 0.19). In contrast, the features circadian movement, transition time, speed moving and time moving indicate no significant correlations with depression. Table 4 summarizes all of the pooled between-person correlations. Feature-specific forest plots can be found in Multimedia Appendix 5.

**Table 4:** Pooled between-person correlations.

Feature	N	Correlation	95% CI	Prediction interval	*I* ^2^
Circadian movement	1201	–0.36	–0.71 to 0.12	–0.91 to 0.64	86%
Distance	1824	–0.25	–0.29 to –0.21	–0.30 to –0.20	0%
Norm entropy	1849	–0.17	–0.29 to –0.04	–0.44 to 0.13	69%
Location variance	2287	–0.17	–0.26 to –0.06	–0.36 to 0.04	58%
Entropy	2254	–0.13	–0.23 to –0.04	–0.35 to 0.10	57%
N cluster	2282	–0.11	–0.18 to –0.03	–0.25 to 0.04	36%
Homestay	2216	0.10	0.00 to 0.19	–0.09 to 0.27	50%
Speed moving	1266	–0.01	–0.07 to 0.06	–0.09 to 0.07	0%
Time moving	748	–0.05	–0.21 to 0.11	–0.45 to 0.36	68%

### Meta-Analysis: Within-Person Correlations

The 3 identified studies reporting on within-person correlations (Table 3) differed widely in the applied methodology of analysis. Saeb et al [34] correlated GPS mobility and activity features with the change in PHQ-9 scores, while Zhang et al [53] used autoregressive models to estimate the correlations over time, and Canzian and Musolesi [57] applied time series analysis to investigate the correlations within each participant across multiple days. Due to this heterogeneity in these studies to derive the reported correlations, we did not perform a meta-analysis for within-person correlations.

### Assessment of Research Standards and Small Study Effects

All included studies (k=19) followed an observational study design. Assessment of the international reporting standards for observational studies revealed that not a single study referenced the international reporting guidelines STROBE. Comparing the k=18 included published studies against the STROBE checklist showed an overall agreement across all items of 73.84% (Stamatis et al [61] excluded from comparison as the provided data originated from additional analyses not covered in that study). Only k=9 (50%) of the studies reported how potential sources of bias were addressed. Regarding missing data, k=6 (33%) reported how missing data was handled and k=3 (17%) reported rates of missingness for the variables of interest. A total of k=7 (39%) of the studies listed reasons for the exclusion of participants at each stage of the study. For all STROBE ratings, please refer to Multimedia Appendix 4.

In addition, neither preregistrations nor study protocols were found for the included studies. A priori power analyses for sample size planning or post hoc for discussion were also not reported in any of the included studies. Power analyses of the included studies indicate that k=16 (84%) studies were sufficiently powered with a power of 80% to detect a correlation of *r*=.50, k=7 (37%) studies for *r*=.30, k=4 (21%) studies for *r*=.20, and k=1 (5%) studies for *r*=.10.

For all mobility and activity features except for time moving, Egger test showed a significant asymmetry in the funnel plot (Table 5). Funnel plots for features with less than k=10 studies, in addition indicate asymmetry. Please refer to Multimedia Appendix 6 for the funnel plots of all between-person features.

**Table 5:** Eggert test for funnel plot asymmetry.

Feature	Intercept	95% CI	*P* value
Homestay	1.55	0.56 to 2.54	<.001
Location variance	–1.44	–2.58 to –0.31	.03
Entropy	–2.11	–2.87 to –1.34	<.001
N clusters	–1.29	–2.22 to –0.35	.02
Norm entropy	–2.30	–3.71 to –1.33	<.001
Distance	1.01	0.46 to 1.56	.01
Time moving	–0.42	–2.74 to 2.66	.98

## Discussion

### Principal Findings

This systematic review with meta-analysis provides pooled correlation estimates for the between-person correlation of GPS-based smart sensing mobility and activity features and depression. We identified robust small to medium between-person correlations for multiple mobility and activity features (ie, distance, normalized entropy, entropy, homestay, number of clusters, and homestay). In contrast to the findings on between-person correlations, this literature for within-person correlations did not allow for meaningful meta-analyses. Furthermore, this study clearly highlights a lack of quality in the literature. While all studies followed an observational study design, not a single study referenced the international reporting guidelines for observational studies (STROBE [38]). Although the reporting in studies addressed STROBE items in most cases (14/19, 74%), deficits were found in addressing sources of bias and missing data. Moreover, we found strong evidence for small study effects potentially indicating publication bias. Most studies were underpowered to detect correlations of the magnitude indicated by the meta-analysis.

Overall, the present analysis highlights the potential of GPS features to infer depression, which is in line with previous published descriptive reviews in the field [26,27,30,32,33]. For instance, with a total sample size of N=1824 and homogenous findings across studies (*I*^2^=0%), distance (*r*=–0.25) might be a promising objective and unobtrusively collectible GPS feature in expert systems for (assisted) diagnoses, just-in-time interventions and personalization of treatment of depression in future [20,65]. Analogously, normalized entropy, entropy, homestay, number of clusters, and homestay were significant markers (*r*=0.10-0.17). However, it is important to note that all studies followed an exploratory design. Both the correlations and the clinical feasibility of such smart sensing augmented expert systems need to be proven in confirmatory trials before clinical applications. The identified pooled correlations and their CI offer a strong foundation for a-priori power analysis to guide confirmatory studies in their study and sample planning [66,67].

However, while some of the investigated correlations are statistically significant and pooled estimates can serve as a basis for future study planning from a statistical point, this does not release from the question of what a clinically relevant correlation is. The process involved in obtaining GPS features (eg, collection of GPS coordinates) as well as the features itself (eg, how much time was spent at home or at work) are highly sensitive. Hence, a discussion taking the clinical significance as well as ethical and privacy issues into account is of utmost importance [20,21,65,68-70]. Furthermore, it needs to be discussed to which extent GPS features as a standalone metric can be used in the diagnostic process or rather as an add on to existing procedures and measurements (eg, patient-reported outcome, ecological momentary assessment, medical record data) [20,24,71]. This discussion would strongly benefit from future studies illuminating these questions from patients’ and health care providers’ (eg, psychotherapists) perspectives.

Another question in the field is, whether GPS features cannot only serve to determine depression from a between-person perspective but also to inform researchers and clinicians about changes within persons (eg, is the increase of time spent at home a reliable indicator for rising depression severity?). This systematic review showed too sparse evidence and too high heterogeneity in applied analyses to run a meta-analysis on the studies conducted so far. Although this study cannot provide answers regarding the robustness across studies and hence evidence for the significance of within-person correlations, in particular, the included study by Zhang et al [53] on the longitudinal relationship of GPS mobility and activity features and depression found higher within-person correlations than between person correlations, underlining the potential benefit of within-person features in clinical applications and the importance of further studies on within-person correlations.

In line with previous reviews on the methods used in smart sensing studies [26,27,31], this study critically highlights gaps in the adherence to international reporting guidelines, potential publication bias, and high heterogeneity in assessment and analysis methodology. Guidelines like STROBE [38] for observational studies and other study design–specific guidelines offer a strong starting point to increase the study and especially reporting quality in the field. However, research on smart sensing and objective sensor data collection comes with its own challenges like (1) high heterogeneity in hardware (eg, used devices) and GPS sampling (eg, many different manufacturers of sensors and variety in the precision of sensing and data quality), (2) a plethora of data preprocessing decisions (eg, sampling rate, outliner detection or feature selection and definition), (3) missing data handling in large fine-grained datasets, and (4) analyses methodology (eg, how to deal with dependencies resulting from multiple measurements from the same individual [24]) [31]. Preregistration and open-access scripts for data preparation and analyses and extensions to existing international guidelines (eg, standards for feature calculation, reporting guidelines, and missing data handling) are highly needed to move towards a standardized and reliable research field.

The current lack of standardization and heterogeneity between studies in assessment frameworks, samples (eg, age and range), sensor sampling rates, devices, feature calculation, methods of handling missing data or accounting for dependencies in the data structure should also be considered when interpreting these findings. Due to the already low number of studies, we were unable to conduct meaningful sensitivity analysis on more homogenous groups of studies (eg, based on data quality, and devices) or meta-regression models to control for other variables (eg, for systematic mobility and activity differences across age groups). As the number of studies and their quality are likely to increase over time, analyses on homogenous groups and meta-regression analyses might become feasible in future. Nevertheless, we want to point out that the heterogeneity for some features (eg, distance) was very low, underlining the robustness of the pooled correlation estimates for these features. Moreover, the weighted pooling in the meta-analysis gave stronger influence on larger studies with more precise estimations of correlations, counteracting the influence of small studies to some extent [41].

Besides analyses on GPS mobility and activity features in more homogenous study groups, other GPS features should be focused on in the future. For instance, GPS sensor data can be used to derive environmental features such as green space, blue space, air pollution, or even regional data like social deprivation scores (eg, zip code based). First studies using smartphone-based GPS collection to infer depression based on such environmental GPS features are promising, and it needs to be investigated to what extent such features show meta-analytically robust correlations [72-74]. Analogously, meta-analytical evidence for other sensors and features is lacking (eg, smartphone screen usage, app usage, and communication and sociability features) and of high importance to inform researchers and practitioners on which objective markers might be suited for depression diagnosis, early-warning, just-in-time interventions, and other clinical applications [21,30-32,65,69,75]. Furthermore, extensions to other prevalent mental disorders (eg, anxiety) and conditions of leading disease burden (eg, pain) are highly needed to investigate the potential of unobtrusive and objective smart sensing data in health care.

### Conclusions

This systematic review and meta-analysis provides evidence for small to medium between-person correlations of GPS mobility and activity features collected through smartphones and depression. In the future, GPS mobility and activity features such as distance may become an important augmentation in the assessment of depression and clinical applications (eg, decision support systems). However, replications in confirmatory studies and improvements in the study quality and standards in the field are needed to draw robust conclusions. Besides, more research on the within-person correlations is necessary to determine the potential of GPS features in not only between-person applications but also from a longitudinal perspective. To fully exploit the potential of smart sensing, further research on associations of depression and other mental disorders with GPS mobility and activity features as well as environmental GPS features (eg, green space) and other sensor modalities (eg, smartphone usage features), and how smart sensing features can be integrated in existing information systems and complex prediction models alongside patient-reported outcomes, clinician ratings, ecological momentary assessments, and medical record data, is of high importance.

## Appendix

Multimedia Appendix 1 PRISMA (Preferred Reporting Items for Systematic Reviews and Meta-Analyses) checklist.

Multimedia Appendix 2 Search terms.

Multimedia Appendix 3 Analysis software.

Multimedia Appendix 4 Dataset and code.

Multimedia Appendix 5 Forest plots.

Multimedia Appendix 6 Funnel plots.

## Abbreviations

AUC: area under the curve
PHQ: Patient Health Questionnaire-9
PRISMA: Preferred Reporting Items for Systematic Reviews and Meta-Analyses
ROC: receiver operating curve
STROBE: Strengthening the Reporting of Observational studies in Epidemiology

## Data Availability

The datasets generated during and/or analyzed during this study and analysis codes are available in the Open Science Framework project (Multimedia Appendix 4)
