# Supplementary material for: The Relation Between Passively Collected GPS Mobility Metrics and Depressive Symptoms: Systematic Review and Meta-Analysis
Source: J Med Internet Res. 2024 Nov 1;26:e51875. doi: 10.2196/51875 (PMC11568401; doi:10.2196/51875)
Supplement: Multimedia Appendix 2 [file jmir_v26i1e51875_app2.docx]

## Multimedia Appendix 2

### Search term

**Embase, Medline, CENTRAL, PsycInfo**

((gps or gis or "geographic* information system" or locat* or position* or passive* sens* or passive* data* or passive* assess* or ambulator* assess* or passive* monitor or ambulator* monitor* or objective behavioral features or track* or daily activ* or physic* activ* or biosens* or bio* sens* or sensing or crowdsens* or smart sens* or smart-sens* or electr* sens* or passive detec* or ambulator* detec* or activ* detec* or mobile sens* or bioinformatic*) and (phone* or smartphone* or "smart phone*" or smart-phone* or mobilephone* or "mobile phone*" or "mobile-phone*" or cellphone* or cell-phone* or "cell phone*" or microcomputer or micro-computer or app or mobile app* or mobile health app* or "smart watch*" or smart-watch* or smartwatch* or wearable* or ambulatory device* or tracking device* or sens* device* or digital phenotyp*) and (mood disorder* or depress* or affective disorder* or dysthym* or anxi* or phobi* or agoraphobi* or panic* or panic disorder* or panic attack*)).ab,kw,ti.

**ACM**

(gps OR "geographic position system" OR gis OR "geographic information system" OR locat* OR position* OR "passive-sensing" OR "passive-sensor" OR "passive data" OR "passive data-collection" OR "passive assess" OR "passive assessment" OR "ambulatory assess" OR "ambulatory assessment" OR "passive monitor" OR "passive monitoring" OR "ambulatory monitor" OR "ambulatory monitoring" OR "objective behavioral features" OR track* OR "daily activity" OR "daily life activity" OR "daily activities" OR "physical activity" OR "physical activities" OR biosens* OR "bio-sensor" OR "biological-sensor" OR "biological-sensing" OR sens* OR crowdsens* OR smartsensing OR smartsensor OR "smart-sensing" OR "smart-sensor" OR "electrical-sensor" OR "electrical-sensing" OR "electric-sensor" OR "electric-sensing" OR "passive detection" OR "passive detector" OR "ambulatory detection" OR "ambulatory detector" OR "activity detection" OR "activity detector" OR "mobile-sensing" OR "mobile-sensor" OR bioinformatic*) AND (phone* OR smartphone* OR "smart phone" OR smart-phone* OR mobilephone* OR "mobile phone" OR mobile-phone* OR cellphone* OR cell-phone* OR "cell phone" OR microcomputer OR micro-computer OR app OR "mobile app" OR "mobile apps" OR "mobile health app" OR "mobile health apps" OR "smart watch" OR smart-watch* OR smartwatch* OR wearable* OR "ambulatory device" OR "ambulatory devices" OR "tracking device" OR "tracking devices" OR "digital phenotyping" OR "digital phenotype") AND ("mood disorder" OR "mood disorders" OR depress* OR "affective disorder" OR "affective disorders" OR dysthym* OR anxi* OR phobi* OR agoraphobi* OR panic* OR "panic-disorder" OR "panic-disorders" OR "panic-attack" OR "panic-attacks" OR ehealth OR telehealth OR mhealth OR telemedicine OR "mobile health" OR "digital health")

**IEEE**

((Document Title:Publication Title:Abstract:IEEE Terms:"gps" OR "geographic position system" OR "gis" OR "geographic information system" OR "locat*" OR "position" OR "passive sensing" OR "passive sensor" OR "passive data" OR "passive data collection" OR "passive assess" OR "passive assessment" OR "ambulatory assess" OR "ambulatory assessment" OR "passive monitor" OR "passive monitoring" OR "ambulatory monitor" OR "ambulatory monitoring" OR "objective behavioral features" OR "track*" OR "daily activity" OR "daily life activity" OR "daily activities" OR "physical activity" OR "physical activities" OR "biosensing" OR "biosensor" OR "biological sensor" OR "biological sensing" OR "sens*" OR "crowdsensing" OR "smartsensing" OR "smartsensor" OR "smart sensing" OR "smart sensor" OR "electrical sensor" OR "electrical sensing" OR "electric sensor" OR "electric sensing" OR "passive detection" OR "passive detector" OR "ambulatory detection" OR "ambulatory detector" OR "activity detection" OR "activitiy detector" OR "mobile sensing" OR "mobile sensor" OR "bioinformatic" OR "bioinformatics") AND (Document Title:Publication Title:Abstract:IEEE Terms:"phone*" OR "smartphone" OR "smart phone" OR "mobilephone" OR "mobile phone" OR "cellphone" OR "cell phone" OR "microcomputer" OR "micro computer" OR "app" OR "apps" OR "mobile application" OR "smart watch" OR "smartwatch" OR "smart watches" OR "smartwatches" OR "wearable*" OR "ambulatory device" OR "ambulatory devices" OR "tracking device" OR "tracking devices" OR "digital phenotyping" OR "digital phenotype") AND (Document Title:Publication Title:Abstract:IEEE Terms:"mood disorder" OR "mood disorders" OR "depress*" OR "affective disorder" OR "affective disorders" OR "dysthymia" OR "anxi*" OR "phobia" OR "phobias" OR "phobic" OR "agoraphobia" OR "panic" OR "panic disorder" OR "panic disorders" OR "panic attack" OR "panic attacks" OR "ehealth" OR "telehealth" OR "mhealth" OR "telemedicine" OR "mobile health" OR "digital health"))

**Web of Science**

(gps OR gis OR "geographic* information system" OR locat* OR position* OR "passive* sens*" OR "passive* data*" OR "passive* assess*" OR "ambulator* assess*" OR "passive* monitor" OR "ambulator* monitor*" OR "objective behavioral features" OR track* OR "daily activ*" OR "physic* activ*" OR biosens* OR "bio* sens*" OR sensing OR crowdsens* OR "smart sens*" OR smart-sens* OR "electr* sens*" OR "passive detec*" OR "ambulator* detec*" OR "activ* detec*" OR "mobile sens*" OR bioinformatic*) AND (phone* OR smartphone* OR "smart phone*" OR smart-phone* OR mobilephone* OR "mobile phone*" OR "mobile-phone*" OR cellphone* OR cell-phone* OR "cell phone*" OR microcomputer OR micro-computer OR app OR "mobile app*" OR "mobile health app*" OR "smart watch*" OR smart-watch* OR smartwatch* OR wearable* OR "ambulatory device*" OR "tracking device*" OR "sens* device*" OR "digital phenotyp*") AND ("mood disorder*" OR depress* OR "affective disorder*" OR dysthym* OR anxi* OR phobi* OR agoraphobi* OR panic* OR "panic disorder*" OR "panic attack*")
