# Supplementary material for: The Relation Between Passively Collected GPS Mobility Metrics and Depressive Symptoms: Systematic Review and Meta-Analysis
Source: J Med Internet Res. 2024 Nov 1;26:e51875. doi: 10.2196/51875 (PMC11568401; doi:10.2196/51875)
Supplement: Multimedia Appendix 3 [file jmir_v26i1e51875_app3.docx]

## Multimedia Appendix 3

### Analysis software

R version 4.2.2 (2022-10-31 ucrt)

Platform: x86_64-w64-mingw32/x64 (64-bit)

Running under: Windows 10 x64 (build 22621)

Matrix products: default

locale:

[1] LC_COLLATE=German_Germany.utf8 LC_CTYPE=German_Germany.utf8 LC_MONETARY=German_Germany.utf8 LC_NUMERIC=C

[5] LC_TIME=German_Germany.utf8

attached base packages:

[1] grid stats graphics grDevices utils datasets methods base

other attached packages:

[1] here_1.0.1 knitr_1.42 esc_0.5.1 metafor_4.2-0 numDeriv_2016.8-1.1 metadat_1.2-0

[7] Matrix_1.5-4 haven_2.5.2 pwr_1.3-0 rockchalk_1.8.157 car_3.1-2 carData_3.0-5

[13] psych_2.3.3 magrittr_2.0.3 readxl_1.4.2 meta_6.2-1 lubridate_1.9.2 forcats_1.0.0

[19] stringr_1.5.0 dplyr_1.1.2 purrr_1.0.1 readr_2.1.4 tidyr_1.3.0 tibble_3.2.1

[25] ggplot2_3.4.2 tidyverse_2.0.0 dmetar_0.0.9000

loaded via a namespace (and not attached):

[1] nlme_3.1-162 rprojroot_2.0.3 prabclus_2.3-2 tools_4.2.2 utf8_1.2.3 R6_2.5.1 colorspace_2.1-0

[8] nnet_7.3-18 withr_2.5.0 netmeta_2.8-2 tidyselect_1.2.0 gridExtra_2.3 mnormt_2.1.1 compiler_4.2.2

[15] cli_3.6.1 xml2_1.3.4 diptest_0.76-0 scales_1.2.1 DEoptimR_1.0-13 robustbase_0.95-1 digest_0.6.31

[22] foreign_0.8-83 minqa_1.2.5 rmarkdown_2.21 pkgconfig_2.0.3 htmltools_0.5.5 MuMIn_1.47.5 sessioninfo_1.2.2

[29] lme4_1.1-33 fastmap_1.1.1 rlang_1.1.1 rstudioapi_0.14 generics_0.1.3 mclust_6.0.0 zip_2.3.0

[36] modeltools_0.2-23 Rcpp_1.0.10 munsell_0.5.0 fansi_1.0.4 abind_1.4-5 lifecycle_1.0.3 stringi_1.7.12

[43] yaml_2.3.7 CompQuadForm_1.4.3 mathjaxr_1.6-0 MASS_7.3-60 plyr_1.8.8 flexmix_2.3-19 parallel_4.2.2

[50] ggrepel_0.9.3 lattice_0.20-45 kutils_1.70 splines_4.2.2 poibin_1.5 hms_1.1.3 pillar_1.9.0

[57] boot_1.3-28 fpc_2.2-10 stats4_4.2.2 magic_1.6-1 glue_1.6.2 evaluate_0.21 vctrs_0.6.2

[64] nloptr_2.0.3 tzdb_0.4.0 cellranger_1.1.0 gtable_0.3.3 kernlab_0.9-32 xfun_0.39 openxlsx_4.2.5.2

[71] xtable_1.8-4 class_7.3-20 cluster_2.1.4 timechange_0.2.0
