# Supplementary material for: The Relation Between Passively Collected GPS Mobility Metrics and Depressive Symptoms: Systematic Review and Meta-Analysis
Source: J Med Internet Res. 2024 Nov 1;26:e51875. doi: 10.2196/51875 (PMC11568401; doi:10.2196/51875)
Supplement: Multimedia Appendix 4 [file jmir_v26i1e51875_app4.docx]

## Multimedia Appendix 4

### Dataset and Code

<https://osf.io/ce45a/>
