# Supplementary material for: The Relation Between Passively Collected GPS Mobility Metrics and Depressive Symptoms: Systematic Review and Meta-Analysis
Source: J Med Internet Res. 2024 Nov 1;26:e51875. doi: 10.2196/51875 (PMC11568401; doi:10.2196/51875)
Supplement: Multimedia Appendix 5 [file jmir_v26i1e51875_app5.docx]

## Multimedia Appendix 5

### Between-person forest plots


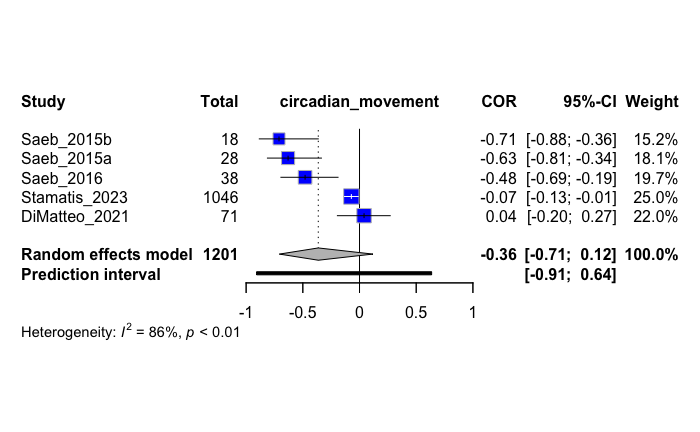


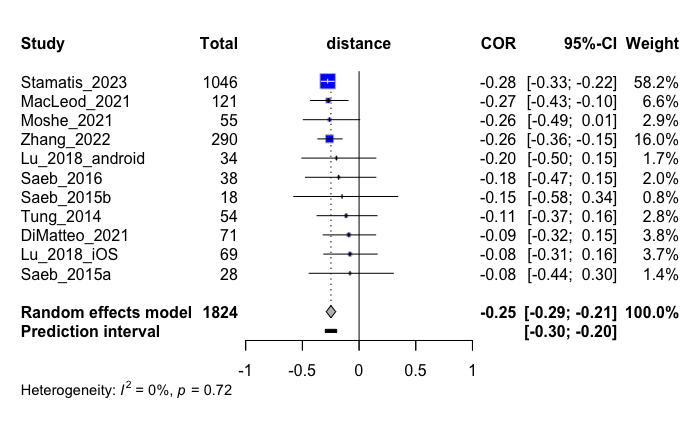


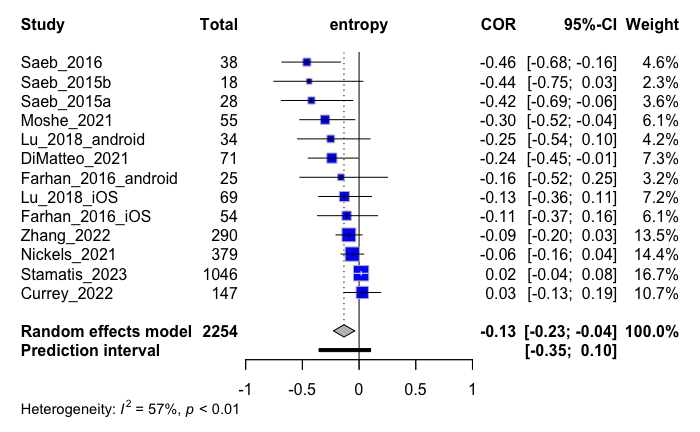


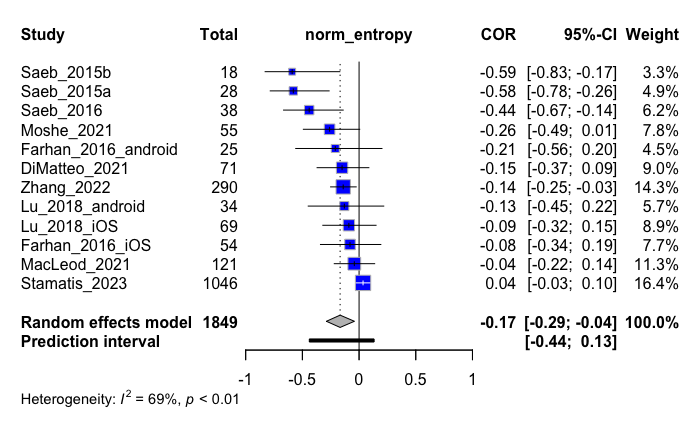


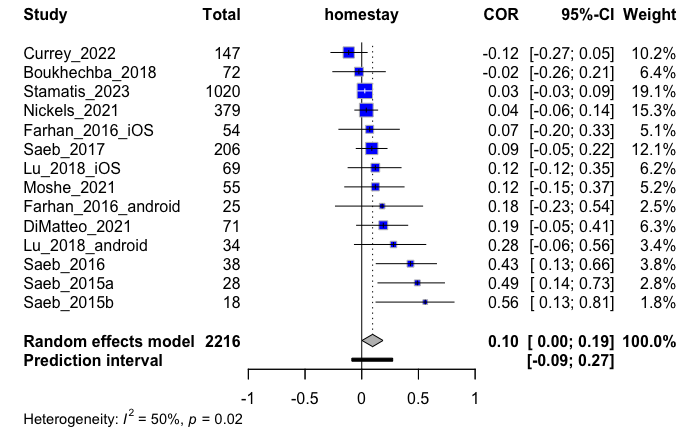


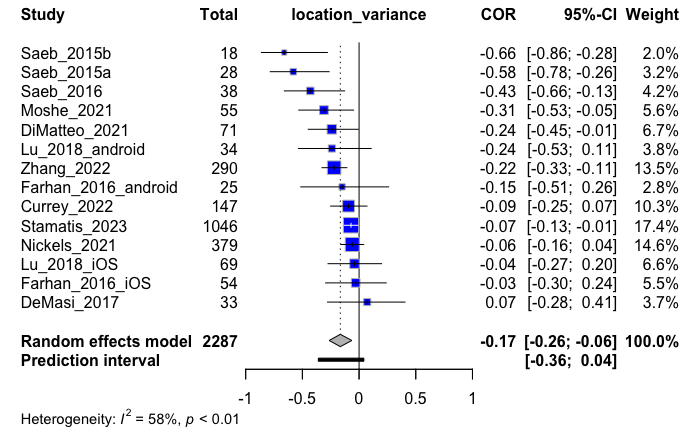


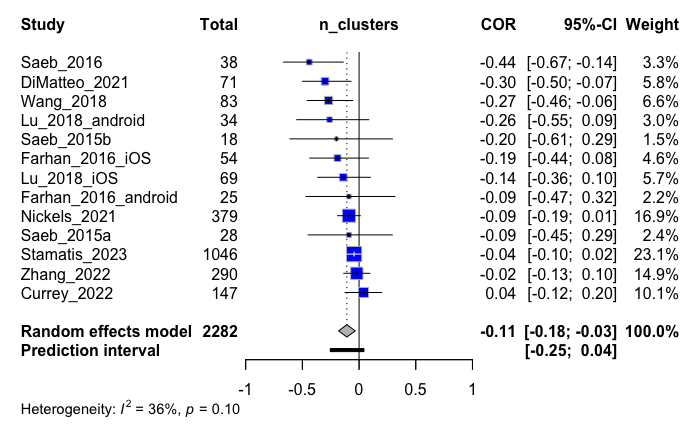


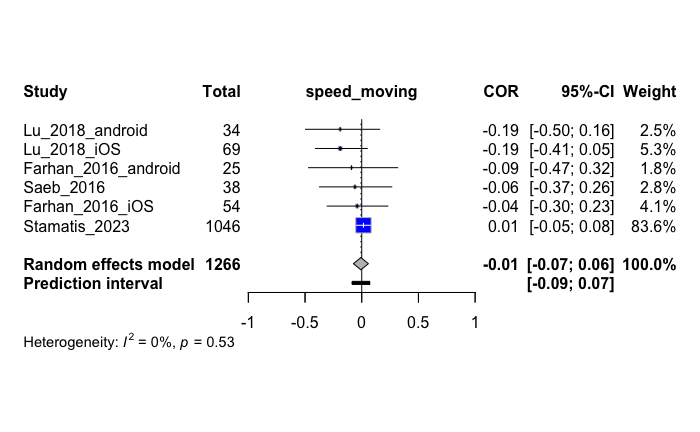


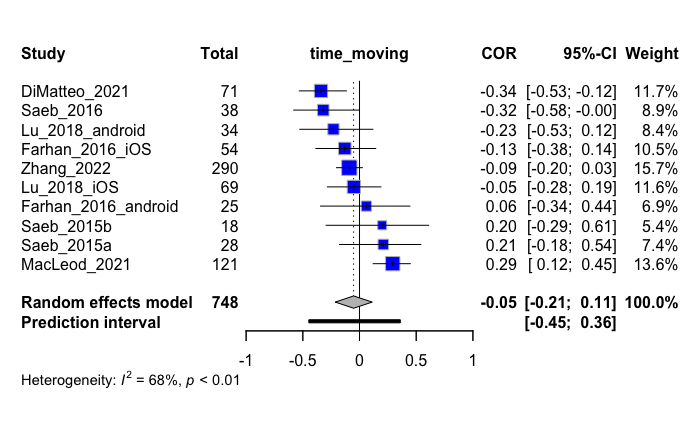


Notes

Saeb_2015a refers to Saeb, S., Zhang, M., Karr, C. J., Schueller, S. M., Corden, M. E., Kording, K. P., & Mohr, D. C. (2015). Mobile Phone Sensor Correlates of Depressive Symptom Severity in Daily-Life Behavior: An Exploratory Study. Journal of Medical Internet Research, 17(7), e175. <https://doi.org/10.2196/jmir.4273>

Saeb_2015b refers to Saeb, S., Zhang, M., Kwasny, M., Karr, C., Kording, K., & Mohr, D. (2015). The Relationship between Clinical, Momentary, and Sensor-based Assessment of Depression. Proceedings of the 9th International Conference on Pervasive Computing Technologies for Healthcare, 3(1), 103–111. <https://doi.org/10.4108/icst.pervasivehealth.2015.259034>
