# Supplementary figures and images for: The Relation Between Passively Collected GPS Mobility Metrics and Depressive Symptoms: Systematic Review and Meta-Analysis
Source: J Med Internet Res. 2024 Nov 1;26:e51875. doi: 10.2196/51875 (PMC11568401; doi:10.2196/51875)

## Multimedia Appendix 6

### Funnel Plots of Between-Person Correlations


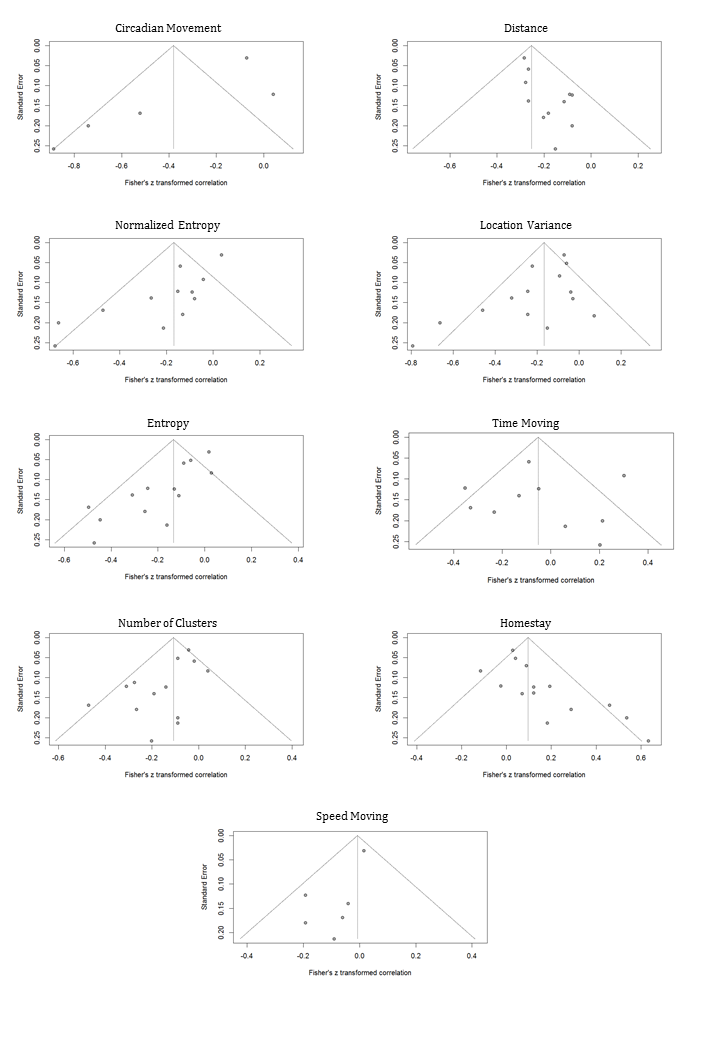

Supplement: Multimedia Appendix 6 [file jmir_v26i1e51875_app6.docx]
